# Species Distribution, Antifungal Susceptibility, and Factors Associated with Recurrence in Vulvovaginal Candidiasis

**DOI:** 10.3390/antibiotics15100969

**Published:** 2026-10-01

**Authors:** Erdal Özmen, Ahmet Cem Yardımcı

**Affiliations:** 1 Department of Obstetrics and Gynecology, VM Medical Park Mersin Hospital, 33200 Mersin, Turkey; 2 Department of Infectious Diseases and Clinical Microbiology, VM Medical Park Mersin Hospital, 33200 Mersin, Turkey; 3 Department of Infectious Diseases and Clinical Microbiology, Faculty of Medicine, Bahçeşehir University, 34734 Istanbul, Turkey

**Keywords:** vulvovaginal candidiasis, *Candida* species, antifungal susceptibility, recurrence, resistance

## Abstract

Objective: Vulvovaginal candidiasis (VVC) is a common condition in clinical practice, and the absence of standardized diagnostic criteria based solely on clinical findings remains a challenge. This study aimed to evaluate the distribution of *Candida* species, antifungal susceptibility patterns, recurrence rates, and clinical factors associated with recurrent disease. Methods: This retrospective study included 345 VVC episodes from 325 patients in whom *Candida* species were isolated from vaginal cultures. Demographic and clinical characteristics, identified *Candida* species, antifungal susceptibility test results, and recurrence patterns were analyzed. Associations with recurrent VVC were evaluated using univariable analyses and multivariable Firth penalized logistic regression. Results: Candida albicans was the most frequently isolated species, accounting for 85.2% (*n* = 294) of cases, followed by *C. glabrata* (9.6%; *n* = 33) and *C. krusei* (1.7%; *n* = 6). Fluconazole susceptibility patterns did not differ significantly between patients with ≥3 recurrent episodes and those with <3 recurrent episodes (*p* = 0.493). Similarly, no significant difference was observed for voriconazole susceptibility (*p* = 0.116). High in vitro susceptibility rates were observed for caspofungin, amphotericin B, and micafungin in both groups. Multivariable Firth logistic regression analysis demonstrated that smoking (OR = 3.83; 95% CI: 1.81–8.81; *p* < 0.001), intrauterine device use (OR = 16.77; 95% CI: 4.13–153.53; *p* < 0.001), and antibiotic use (OR = 7.81; 95% CI: 3.90–17.30; *p* < 0.001) were significantly associated with recurrence, whereas pregnancy was not significantly associated with recurrence (OR = 2.05; 95% CI: 0.78–5.66; *p* = 0.147).

## 1. Introduction

Vulvovaginal candidiasis is a frequent fungal infection of the female genital tract and remains a leading cause of vaginitis among women of reproductive age, second only to bacterial vaginosis [1,2,3]. In Turkey, the prevalence of VVC is 11–28.9%. *Candida albicans* is isolated from 80–90% of patients who experience acute VVC [4].

Candida albicans remains the predominant pathogen, although infections caused by non-albicans *Candida* species have increased over recent years. These species may exhibit different antifungal susceptibility profiles and are frequently associated with recurrent disease [5].

The frequent occurrence of vulvovaginal candidiasis and the lack of diagnostic criteria in the literature, consisting of history and physical examination findings for VVC, and experience on species, represent unmet needs in this field. Within the scope of this research, we aimed to elucidate the distribution of *Candida* species, analyze antifungal susceptibility patterns, determine recurrence rates, and identify possible risk factors associated with these recurrences. These data will contribute to understanding the epidemiology of vulvovaginal candidiasis in our region, reviewing empirical treatment options, and monitoring antifungal resistance.

Despite extensive research on *Candida* species distribution and antifungal susceptibility, data focusing on the relative contribution of clinical risk factors and microbiological susceptibility patterns to recurrent disease remain limited. The relative associations of antifungal susceptibility patterns and host-related clinical factors with recurrence remain incompletely defined. Therefore, this study aimed to evaluate clinical and microbiological factors associated with recurrent vulvovaginal candidiasis in a real-world clinical setting.

## 2. Method

This retrospective study included 345 VVC episodes from 325 patients who presented to our institution between 1 January 2022 and 31 December 2025, and in whom Candida spp. growth was detected in vaginal cultures. The demographic and clinical characteristics of the patients, the isolated *Candida* species, antifungal susceptibility test results, and the number and characteristics of recurrent cases were analyzed. The unit of analysis was the VVC episode/isolate, and repeated presentations from the same patient were retained as separate episodes.

All procedures were conducted in accordance with the ethical standards of the responsible committee on human experimentation (institutional and national) and with the Declaration of Helsinki of 1975, as revised in 2008. Ethical approval was obtained from the Toros University Scientific Research and Publication Ethics Committee (Approval date: 22 September 2025; Decision No: 2025-07/141). This retrospective study was based exclusively on anonymized routinely collected clinical records. No patients were recruited prospectively, and no interventions were performed for research purposes. Data extraction and statistical analyses were initiated only after ethical approval had been obtained. Because of the retrospective design and the use of anonymized routinely collected clinical data, the requirement for informed consent was waived by the ethics committee.

### 2.1. Inclusion Criteria

Women aged ≥ 18 years presenting with clinical findings compatible with vulvovaginal candidiasis (including vaginal discharge, pruritus, burning sensation, or vulvovaginal erythema) together with positive vaginal culture for *Candida* spp. were eligible for inclusion. Women with positive vaginal cultures in the absence of compatible clinical symptoms were considered to have *Candida* colonization rather than infection and were therefore excluded.

### 2.2. Exclusion Criteria

Patients with missing clinical or laboratory data, cases referred from another center during the study period for whom complete clinical data were unavailable, and immunocompromised patients were excluded. Immunocompromised patients were defined as those with human immunodeficiency virus (HIV) infection, solid organ or hematopoietic stem cell transplantation, active malignancy receiving chemotherapy, treatment with biological immunosuppressive agents, systemic corticosteroid therapy equivalent to ≥20 mg/day of prednisolone for ≥14 days, or primary immunodeficiency disorders. Eligibility was determined from routinely available medical records. No additional laboratory screening (including routine HIV testing) was performed solely for research purposes.

### 2.3. Definition of Recurrence

Definitions of recurrent vulvovaginal candidiasis (RVVC) vary among international guidelines. For the purposes of this study, RVVC was defined as ≥3 clinically documented symptomatic episodes within 12 months, consistent with the 2021 CDC Sexually Transmitted Infections Treatment Guidelines and several contemporary studies. Previous episodes were retrospectively identified from the patients’ medical records. To ensure diagnostic accuracy, each recurrent episode was required to meet all of the following criteria:

1. Clinical symptoms compatible with vulvovaginal candidiasis;
2. Isolation of *Candida* spp. by positive vaginal culture.

Episodes caused by different *Candida* species were also considered recurrent VVC, provided that each episode fulfilled the predefined clinical and microbiological diagnostic criteria.

### 2.4. Microbiological Procedures

Vaginal swab specimens were transported immediately to the microbiology laboratory and inoculated onto Sabouraud Dextrose Agar (RTA, Kocaeli, Türkiye). The inoculated media were incubated at 35 °C under aerobic conditions according to routine laboratory procedures. *Candida* species identification was performed using the VITEK^®^ 2 automated system (bioMérieux, Marcy-l’Étoile, France) with the VITEK^®^ 2 YST identification card, and antifungal susceptibility testing was performed using the VITEK^®^ 2 AST-YS08 susceptibility testing card.

Antifungal susceptibility results were interpreted according to the EUCAST clinical breakpoint tables applicable at the time of testing. Because EUCAST breakpoint tables were updated during the study period (2022–2025), results were classified according to the version in use at the time of testing rather than retrospectively applying a single current version. EUCAST version 11.0 was used from 2 December 2024. The antifungal agents evaluated included fluconazole, voriconazole, amphotericin B, caspofungin, and micafungin/anidulafungin.

Categorical results were reported only when an applicable species–drug breakpoint was available. Susceptibility percentages were calculated using categorically interpretable S, R, and, where applicable, I/SDD results. TRM results and isolates with only numerical MIC values without categorical interpretation were excluded from percentage calculations and group comparisons. The microbiology laboratory participated in an external quality assessment program (LabPT, Ankara, Türkiye) for microbiological identification during the study period.

### 2.5. Empirical Treatment Approach

Throughout the study period, uncomplicated vulvovaginal candidiasis was primarily treated with oral fluconazole in accordance with contemporary clinical practice guidelines. Topical azole agents (including clotrimazole and miconazole vaginal formulations) were used as alternative or adjunctive therapy, particularly in patients with mild-to-moderate disease, contraindications to systemic treatment, or pregnancy.

### 2.6. Variables Assessed

The following variables were evaluated for each patient: demographic characteristics, predefined clinical risk factors, isolated *Candida* species, antifungal susceptibility patterns, and the occurrence of recurrent vulvovaginal candidiasis.

Smoking was defined as documented tobacco use recorded in the medical record at the time of diagnosis. Antibiotic use was defined as documented administration of systemic antibacterial therapy within the 3 months preceding the diagnosis; antifungal agents were not included in this category. The dedicated antibiotic-use variable in the clinical records was used for all analyses reported in the manuscript. Intrauterine device (IUD) use was defined as the presence of an IUD at the time of diagnosis. Pregnancy was defined as a documented ongoing pregnancy at the time of presentation.

The presence of clinical risk factors was defined as the presence of at least one of the following conditions: smoking, recent antibiotic use, intrauterine device use, pregnancy, or other documented comorbidities.

### 2.7. Statistical Analysis

Patient data collected within the scope of the study were analyzed using IBM Statistical Package for the Social Sciences (SPSS) for Windows, version 26.0 (IBM Corp., Armonk, NY, USA). Continuous variables were assessed for normality and are presented as mean ± standard deviation and minimum–maximum values, while categorical variables are expressed as numbers and percentages.

The Chi-square test or Fisher’s exact test, as appropriate, was used for intergroup comparisons of categorical variables. The primary outcome of the study was recurrent vulvovaginal candidiasis, defined as ≥3 episodes per year. Associations between clinical variables and recurrent episodes were evaluated using univariate analyses and, because of sparse cells and the risk of separation, multivariable Firth penalized logistic regression. Odds ratios (ORs) with 95% confidence intervals (CIs) were calculated to quantify the strength of associations. All statistical tests were two-sided, and a *p* value < 0.05 was considered statistically significant.

## 3. Results

A total of 345 VVC episodes from 325 patients were included in the study. The mean age of the patients was 33.7 ± 7.2 years (range, 18–52 years). At least one risk factor was identified in 41.7% (*n* = 144) of the episodes, while 58.3% (*n* = 201) had no risk factors. The most frequently reported risk factors were antibiotic use (22.0%; *n* = 76), smoking (14.5%; *n* = 50), intrauterine device (IUD) use (7.0%; *n* = 24), and pregnancy (4.9%; *n* = 17), respectively. Other risk factors included hypothyroidism (0.6%; *n* = 2), diabetes (0.6%; *n* = 2), Mediterranean anemia/thalassemia carrier status (0.3%; *n* = 1), and insulin resistance (0.3%; *n* = 1) at low rates. The analysis revealed that 57.7% (*n* = 199) of the episodes were classified as recurrent VVC (≥3 episodes/year). The most frequently isolated species in microbiological examination was *Candida albicans*, detected in 85.2% (*n* = 294). This was followed by *C. glabrata* (9.6%; *n* = 33) and *C. krusei* (1.7%; *n* = 6). Other *Candida* species were detected at lower frequencies (Table 1).

In antifungal susceptibility analysis, 68.6% of the strains were susceptible to fluconazole, 16.9% were resistant, and 14.5% were susceptible at increased exposure (I). When voriconazole susceptibility was assessed, the rates of susceptible and resistant strains were similar (42.7% and 43.0%, respectively), while 14.3% were susceptible at increased exposure (I). Susceptibility rates for caspofungin and micafungin were high, at 98.8% and 97.6%, respectively. Amphotericin B susceptibility was 93.3%, with resistance observed in 6.7%. The distribution of antifungal susceptibility across the *Candida* species is presented in Table 2. Antifungal susceptibility percentages were calculated based only on categorically interpretable isolates (S + R + I/SDD), excluding technically unreportable results and numerical MIC values without categorical interpretation; TRM results were reported separately.

For fluconazole, 78.7% of *C. albicans* isolates were susceptible, 13.7% were resistant, and 7.6% were susceptible at increased exposure (I). No fluconazole susceptibility was observed among *C. glabrata* isolates; 79.3% were classified as susceptible dose-dependent (SDD), and 20.7% were resistant. All *C. krusei* isolates were resistant to fluconazole. Fluconazole susceptibility rates varied among other *Candida* species. For voriconazole, 40.1% susceptibility, 44.9% resistance, and 15.0% susceptibility at increased exposure (I) were observed among *C. albicans* isolates. For *C. glabrata*, 32 isolates had only numerical voriconazole MIC values without categorical interpretation and were therefore excluded from categorical percentage calculations; only one isolate had a categorical result and was classified as resistant. High susceptibility rates were observed across *Candida* species to the echinocandin antifungals caspofungin and micafungin. Overall, caspofungin susceptibility was 98.8%, whereas micafungin susceptibility was 97.6%. Resistance was observed in only a limited number of *C. albicans* and *C. lusitaniae* isolates. For amphotericin B, the overall susceptibility rate was 93.3%, with resistance detected in 6.7%.

When the relationship between recurrent episode frequency (≥3/year) and the presence of *Candida* species was evaluated, a significant difference in species distribution was observed between the two groups. The prevalence of *C. albicans* was 95.0% (*n* = 189) in the ≥3 episodes group, while it was 71.9% (*n* = 105) in the <3 episodes group (*p* < 0.001). In contrast, *C. glabrata* was observed at a higher rate in the <3 episodes group (16.4% vs. 4.5%; *p* < 0.001). *C. krusei* and *C. lusitaniae* were detected only in the <3 episodes group, and the difference between the groups was statistically significant for both species (*p* = 0.005 and *p* = 0.031, respectively). No significant association was found between recurrence status and other *Candida* species (Table 3).

When antifungal susceptibility results were compared according to recurrence status, the distributions of fluconazole susceptibility categories were similar between the ≥3 recurrent episodes and <3 recurrent episodes groups (*p* = 0.493). Similarly, the distribution of voriconazole susceptibility did not differ significantly between the two groups (*p* = 0.116). High susceptibility rates were observed in both groups for caspofungin, amphotericin B, and micafungin. No statistically significant difference was found between recurrence status and susceptibility distribution for these antifungals (*p* = 1.000, *p* = 1.000, and *p* = 0.726, respectively).

In the univariate logistic regression analysis evaluating factors associated with recurrence (≥3 episodes/year), smoking, IUD use, and antibiotic use were significantly associated with recurrence. Smoking was associated with higher odds of recurrence (OR = 3.95; 95% CI: 1.85–8.42; *p* < 0.001), as were IUD use (OR = 18.95; 95% CI: 2.53–142.01; *p* < 0.001) and antibiotic use (OR = 7.73; 95% CI: 3.70–16.13; *p* < 0.001). No significant associations were observed for pregnancy (OR = 1.05; 95% CI: 0.39–2.82; *p* = 0.925) or diabetes mellitus (OR = 0.73; 95% CI: 0.05–11.81; *p* = 0.826).

In the multivariable Firth penalized logistic regression analysis, smoking (adjusted OR = 3.83; 95% CI: 1.81–8.81; *p* < 0.001), IUD use (adjusted OR = 16.77; 95% CI: 4.13–153.53; *p* < 0.001), and antibiotic use (adjusted OR = 7.81; 95% CI: 3.90–17.30; *p* < 0.001) remained significantly associated with recurrence. Pregnancy was not significantly associated with recurrence in the multivariable model (adjusted OR = 2.05; 95% CI: 0.78–5.66; *p* = 0.147) (Table 4).

## 4. Discussion

Vulvovaginal candidiasis (VVC) is the second most common vaginal infection in women after bacterial vaginosis. *Candida* species are present in the vagina as both colonizers and pathogens. Studies have shown that *Candida* colonization can last for months or years. As no single clinical feature is diagnostic of vulvovaginal candidiasis, confirmation by microbiological testing is essential for accurate diagnosis [6].

Despite the availability of alternative antifungal agents, fluconazole continues to be widely used as first-line therapy for vulvovaginal candidiasis. Azole antifungals remain the cornerstone of treatment for uncomplicated VVC [7]. Because non-*albicans Candida* species are frequently less susceptible to azoles, treatment may be more challenging, and approximately 50% of affected women may present with few or no symptoms [8]. Diabetes mellitus is a recognized risk factor for complicated VVC, and infections caused by non-*albicans Candida* species—particularly *C. glabrata*—are more common in these patients and are associated with poorer responses to antifungal therapy [9]. Current guidelines recommend topical azole therapy for the treatment of VVC during pregnancy [8,9,10]. In our study, the most frequently reported risk factors were antibiotic use (22.0%), smoking (14.5%), intrauterine device (IUD) use (7.0%), and pregnancy (4.9%). Diabetes mellitus was identified in only 0.6% of episodes.

Recurrent vulvovaginal candidiasis (RVVC) remains a chronic and challenging condition affecting women worldwide. Definitions of RVVC vary among international guidelines. The 2021 CDC Sexually Transmitted Infections Treatment Guidelines define RVVC as three or more symptomatic episodes within 12 months, whereas the 2022 European expert panel generally defines RVVC as four or more episodes annually [11,12]. For consistency with our study design, RVVC was defined as ≥3 symptomatic episodes within one year. RVVC is a multifactorial condition that may involve both Candida albicans and non-albicans *Candida* species, the latter often demonstrating reduced susceptibility to azole antifungal agents [13]. Azole agents remain the mainstay of treatment for VVC and RVVC [7,11]. Fluconazole resistance may occur in Candida isolates causing RVVC, particularly after repeated or prolonged azole exposure [12]. Our antifungal susceptibility analysis showed that 68.6% of isolates were susceptible to fluconazole, 16.9% were resistant, and 14.5% showed susceptibility at increased exposure (I). Voriconazole susceptibility was heterogeneous, with comparable proportions of susceptible and resistant isolates. In vitro susceptibility to echinocandins was high in the tested isolates. However, echinocandins are not standard first-line therapy for vulvovaginal candidiasis and are generally administered intravenously; therefore, the echinocandin results in this study should be interpreted as microbiological susceptibility data rather than as evidence supporting their routine clinical use for VVC. Their inclusion provides a broader description of the in vitro antifungal susceptibility profile of the isolates, particularly in the context of azole nonsusceptibility. Amphotericin B susceptibility exceeded 90%. Among *C. albicans* isolates, fluconazole susceptibility remained relatively high, although resistance and susceptibility at increased exposure (I) were not negligible. Recurrence was significantly associated with smoking, IUD use, and antibiotic exposure, whereas antifungal susceptibility patterns did not differ significantly according to recurrence status. These observational findings do not establish a causal relationship. The relatively high nonsusceptibility rates observed for some antifungal agents, particularly voriconazole, should be interpreted cautiously because susceptibility testing was performed using an automated system (VITEK^®^ 2) rather than a reference broth microdilution method. Differences between automated and reference methods may affect susceptibility categorization for certain species–drug combinations. Confirmation using a reference method would strengthen the interpretation of these findings.

While *C. albicans* remains the predominant species causing VVC, a shift toward non-albicans Candida (NAC) species has been reported in recent years, with NAC species accounting for approximately 10–45% of cases in some studies. Treatment failures may occur more frequently in infections caused by NAC species because of intrinsic or acquired resistance and reduced susceptibility to some antifungal agents [14]. NAC species have also been associated with reduced azole susceptibility and, in some studies, higher recurrence rates, whereas most *C. albicans* isolates generally remain susceptible to azole antifungals [15,16,17,18,19,20,21,22,23]. Consistent with these findings, *C. albicans* was the predominant species in our cohort. However, *C. glabrata* was more frequently identified in the <3 episodes/year group, while *C. krusei* and *C. lusitaniae* were detected only in this group. Thus, our findings do not support a general association between NAC species and increased recurrence.

Previous studies have identified several host-related factors associated with recurrent VVC, including antibiotic exposure, oral contraceptive use, vaginal hygiene practices, intrauterine device use, allergic diseases, obesity, and probiotic use [16,17,19,23,24]. However, reported risk factors have varied across studies. Consistent with the existing literature, smoking, antibiotic exposure, and intrauterine device use were significantly associated with recurrent VVC in the multivariable analysis, whereas pregnancy was not significantly associated with recurrence. Diabetes mellitus was also not significantly associated with recurrence in the univariate analysis, although the very small number of cases limited further evaluation.

From a clinical perspective, the observed associations suggest that host-related clinical factors may be relevant to recurrence; however, the retrospective design does not permit causal inference, and these findings require confirmation in prospective studies. Routine escalation of antifungal therapy without considering potentially modifiable clinical factors may be insufficient in the management of recurrent disease.

In this retrospective cohort, smoking, intrauterine device use, and recent antibiotic exposure were associated with recurrent vulvovaginal candidiasis. Candida albicans was the most frequently isolated species, followed by *C. glabrata* and *C. krusei*. Among the antifungal agents tested, high in vitro susceptibility rates were observed for caspofungin and micafungin, followed by amphotericin B and fluconazole; however, echinocandins are not standard first-line therapy for VVC, and these results should not be interpreted as a treatment recommendation. Overall, the findings support consideration of potentially modifiable host-related factors together with appropriate microbiological evaluation. Because of the retrospective observational design, the reported associations should not be interpreted as causal relationships.

This study has several limitations. First, its retrospective observational design permits identification of associations but does not establish causality, and certain clinical variables, including smoking status and antibiotic exposure, may have been incompletely documented. Second, antifungal susceptibility testing was performed using an automated system rather than a reference broth microdilution method, which may have influenced susceptibility categorization for some isolates. Because the study spanned 2022–2025, during which EUCAST breakpoint tables were updated, the analysis relied on categorical susceptibility interpretations documented in the laboratory records rather than retrospectively imposing one current breakpoint table across the entire study period. Third, data regarding prior antifungal treatment history were not consistently available and therefore could not be analyzed. Finally, several potentially relevant variables, including body mass index, menopausal status, sexual behavior, vaginal hygiene practices, vaginal douching, oral contraceptive use, probiotic use, glycemic control, and vaginal microbiota composition, were not routinely available in the medical records and therefore could not be included in the analyses. These limitations should be considered when interpreting the findings.

## Figures and Tables

**Table 1:** Baseline demographic and clinical characteristics of the study population.

N = 345			
Age Mean ± SD (Min–Max)		33.7 ± 7.2 (18–52)	
		*n*	%
Risk Factors	Yes	144	41.7%
	No	201	58.3%
Common Risk Factors	Smoking	50	14.5%
	Antibiotics	76	22.0%
	Intrauterine device (IUD)	24	7%
	Pregnancy	17	4.9%
	Diabetes	2	0.6%
	Hypothyroidism	2	0.6%
	Mediterranean anemia/thalassemia carrier	1	0.3%
	Insulin resistance	1	0.3%
Recurrent episodes ≥ 3/year	Yes	199	57.7%
	No	146	42.3%
*Candida* species	*C. albicans*	294	85.2%
	*C. glabrata*	33	9.6%
	*C. krusei*	6	1.7%
	*C. lusitaniae*	4	1.2%
	*C. kefyr*	2	0.6%
	*C. spherica*	2	0.6%
	*C. tropicalis*	2	0.6%
	*C. dubliniensis*	1	0.3%
	*C. parapsilosis*	1	0.3%
Fluconazole Sensitivity	S	203	68.6%
	R	50	16.9%
	I	43	14.5%
Voriconazole Sensitivity	S	125	42.7%
	R	126	43.0%
	I	42	14.3%
	TRM	5	
Caspofungin Susceptibility	S	325	98.8%
	R	4	1.2%
Amphotericin B Sensitivity	S	304	93.3%
	R	22	6.7%
	TRM	4	
Micafungin Sensitivity	S	321	97.6%
	R	8	2.4%

Risk factors were not mutually exclusive; therefore, individual risk-factor counts may overlap.

**Table 2:** Distribution of Antifungal Susceptibility According to *Candida* Species.

*Candida* Species	Fluconazole S/R/I *n* (%)	Voriconazole		Caspofungin S/R *n* (%)	Amphotericin B		Micafungin S/R *n* (%)
		S/R/I *n* (%)	TRM *n*		B S/R *n* (%)	TRM *n*	
*C. albicans*	S: 196 (78.7)/R: 34 (13.7)/I: 19 (7.6)	S: 110 (40.1)/R: 123 (44.9)/I: 41 (15.0)	5	S: 275 (98.9)/R: 3 (1.1)	S: 254 (92.4)/R: 21 (7.6)	4	S: 271 (97.5)/R: 7 (2.5)
*C. glabrata*	S: 0 (0.0)/R: 6 (20.7)/SDD: 23 (79.3)	S: 0/R: 1/I: 0	0	S: 33 (100)/R: 0 (0.0)	S: 33 (100)/R: 0 (0.0)	0	S: 33 (100)/R: 0 (0.0)
*C. krusei*	S: 0 (0.0)/R: 6 (100)/I: 0 (0.0)	S: 6 (100)/R: 0 (0.0)/I: 0 (0.0)	0	S: 6 (100)/R: 0 (0.0)	S: 6 (100)/R: 0 (0.0)		S: 6 (100)/R: 0 (0.0)
*C. lusitaniae*	S: 3 (75.0)/R: 1 (25.0)/I: 0 (0.0)	S: 3 (75.0)/R: 1 (25.0) /I: 0 (0.0)	0	S: 3 (75.0)/R: 1 (25.0)	S: 3 (75.0)/R: 1 (25.0)	0	S: 3 (75.0)/R: 1 (25.0)
*C. kefyr*	S: 0 (0.0)/R: 2 (100)/I: 0 (0.0)	S: 2 (100)/R: 0 (0.0)/I: 0 (0.0)	0	S: 2 (100)/R: 0 (0.0)	S: 2 (100)/R: 0 (0.0)	0	S: 2 (100)/R: 0 (0.0)
*C. spherica*	S: 1 (50.0)/R: 0 (0.0)/I: 1 (50.0)	S: 1 (50.0)/R: 0 (0.0)/I: 1 (50.0)	0	S: 2 (100)/R: 0 (0.0)	S: 2 (100)/R: 0 (0.0)	0	S: 2 (100)/R: 0 (0.0)
*C. tropicalis*	S: 2 (100)/R: 0 (0.0)/I: 0 (0.0)	S: 2 (100)/R: 0 (0.0)/I: 0 (0.0)	0	S: 2 (100)/R: 0 (0.0)	S: 2 (100)/R: 0 (0.0)	0	S: 2 (100)/R: 0 (0.0)
*C. dubliniensis*	S: 0 (0.0)/R: 1 (100)/I: 0 (0.0)	S: 0 (0.0)/R: 1 (100)/I: 0 (0.0)	0	S: 1 (100)/R: 0 (0.0)	S: 1 (100)/R: 0 (0.0)	0	S: 1 (100)/R: 0 (0.0)
*C. parapsilosis*	S: 1 (100)/R: 0 (0.0)/I: 0 (0.0)	S: 1 (100)/R: 0 (0.0)/I: 0 (0.0)	0	S: 1 (100)/R: 0 (0.0)	S: 1 (100)/R: 0 (0.0)	0	S: 1 (100)/R: 0 (0.0)
General	S: 203 (68.6)/R: 50 (16.9)/I: 43 (14.5)	S: 125 (42.7)/R: 126 (43.0)/I: 42 (14.3)	5	S: 325 (98.8)/R: 4 (1.2)	S: 304 (93.3)/R: 22 (6.7)	4	S: 321 (97.6)/R: 8 (2.4)

Percentages were calculated using only isolates with categorically interpretable susceptibility results for each species–antifungal combination. S denotes susceptible, R resistant, I susceptible at increased exposure, and SDD susceptible dose-dependent. TRM results and numerical MIC values without categorical interpretation were excluded from percentage calculations and comparative analyses. For *C. glabrata*, voriconazole results for 32 isolates were available only as numerical MIC values without categorical interpretation and were therefore excluded from these calculations.

**Table 3:** *Candida* Species and Antifungal Susceptibility Results Based on Recurrence Rate (≥3/year).

		Recurrent Episodes		
		≥3 Episodes *n* (%)	<3 Episodes *n* (%)	*p*-Value
*Candida* Species Growth	*C. albicans*	189 (95)	105 (71.9)	<0.001
	*C. glabrata*	9 (4.5)	24 (16.4)	<0.001
	*C. krusei*	0 (0.0)	6 (4.1)	0.005
	*C. lusitaniae*	0 (0.0)	4 (2.7)	0.031
	*C. kefyr*	0 (0.0)	2 (1.4)	0.178
	*C. spherica*	0 (0.0)	2 (1.4)	0.178
	*C. tropicalis*	0 (0.0)	2 (1.4)	0.178
	*C. dubliniensis*	1 (0.5)	0 (0.0)	1.000
	*C. parapsilosis*	0 (0.0)	1 (0.7)	0.423
Fluconazole susceptibility	S	124 (71.3)	79 (64.8)	0.493
	R	27 (15.5)	23 (18.9)	
	I/SDD	23 (13.2)	20 (16.4)	
Voriconazole susceptibility	S	68 (38.0)	57 (50.0)	0.116
	R	82 (45.8)	44 (38.6)	
	I	29 (16.2)	13 (11.4)	
Caspofungin susceptibility	S	188 (98.9)	137 (98.6)	1.000
	R	2 (1.1)	2 (1.4)	
Amphotericin B susceptibility	S	175 (93.1)	129 (93.5)	1.000
	R	13 (6.9)	9 (6.5)	
Micafungin susceptibility	S	186 (97.9)	135 (97.1)	0.726
	R	4 (2.1)	4 (2.9)	

*Candida* species were compared individually using Fisher’s exact test. Pearson’s chi-square test was used for fluconazole and voriconazole susceptibility distributions, whereas Fisher’s exact test was used for the other antifungal agents. Percentages were calculated based on evaluable susceptibility results within each recurrence group.

**Table 4:** Univariate and multivariable analyses of factors associated with recurrent VVC (≥3 episodes/year).

	Univariate			Multivariable *		
	OR	95% CI (Lower–Upper)	*p*-Value	OR	95% CI (Lower–Upper)	*p*-Value
Smoking	3.95	1.85–8.42	<0.001	3.83	1.81–8.81	<0.001
IUD use	18.95	2.53–142.01	<0.001	16.77	4.13–153.53	<0.001
Pregnancy	1.05	0.39–2.82	0.925	2.05	0.78–5.66	0.147
Antibiotic use	7.73	3.70–16.13	<0.001	7.81	3.90–17.30	<0.001
Diabetes mellitus	0.73	0.05–11.81	0.826			

* Multivariable analysis was performed using Firth penalized logistic regression because of sparse cells. The model included smoking, IUD use, pregnancy, and antibiotic use. Antibiotic use was defined using the dedicated antibiotic-use variable. Diabetes mellitus was not included in the multivariable model because of the very small number of cases (*n* = 2). OR, odds ratio; CI, confidence interval.

## Data Availability

The data supporting this study’s findings are available on request from the corresponding author. The data are not publicly available due to privacy or ethical restrictions.
